# From Purpura to pulmonary hemorrhage: dermatological clues to systemic vasculitis – a case of granulomatosis with polyangiitis

**DOI:** 10.1097/MS9.0000000000005297

**Published:** 2026-07-02

**Authors:** Naqeeb Ullah, Azra Jabin, Huma Isar, Samreen Javed, Farah Gul, Filza Ajmal, Sundas Safdar, Hanif Ullah Hanfi, Ramin Khan, Adnan Ayub, Samim Noori, Sardar Noman Qayyum

**Affiliations:** a Department of Medicine, Lady Reading Hospital, Peshawar, Pakistan; b Department of Medicine, Khyber Medical College, Peshawar, Pakistan; c Department of Medicine, Hayatabad Medical Complex, Peshawar, Pakistan; d Department of Medicine, Mardan Medical Complex, Mardan, Pakistan; e Department of Medicine, Bacha Khan Medical College, Mardan, Pakistan; f Department of Medicine, Medical University Pleven, Pleven, Bulgaria; g Faculty of Medicine, Nangarhar University, Nangarhar, Afghanistan

**Keywords:** ANCA-associated vasculitis, cutaneous ulcers, direct immunofluorescence, granulomatosis with polyangiitis, IgA vasculitis, pauci-immune vasculitis, pulmonary hemorrhage

## Abstract

**Introduction:**

Granulomatosis with polyangiitis (GPA) is a rare ANCA-associated vasculitis that can convincingly mimic IgA vasculitis when it presents with palpable purpura and a leukocytoclastic biopsy. Failure to perform direct immunofluorescence (DIF) and ANCA testing at presentation is a recognized cause of diagnostic delay and irreversible organ damage.

**Case presentation:**

A 47-year-old woman, previously labeled as IgA vasculitis on the basis of a leukocytoclastic skin biopsy without DIF, returned 1 year later with recurrent necrotizing leg ulcers, ENT involvement, and hemoptysis. Strong c-ANCA/PR3 positivity (>100 U/mL), microscopic hematuria, an HRCT pattern of diffuse alveolar hemorrhage, and a repeat skin biopsy demonstrating pauci-immune necrotizing leukocytoclastic vasculitis (DIF negative for IgA) confirmed GPA. Pulse methylprednisolone, followed by oral corticosteroids and IV cyclophosphamide, produced complete reversal of pulmonary hemorrhage within 2 weeks and progressive healing of cutaneous ulcers.

**Discussion:**

Persistent or recurrent cutaneous vasculitis – particularly necrotizing ulcers in an adult – should prompt ANCA testing and DIF, even when an initial diagnosis of IgA vasculitis seems plausible. Pauci-immune histology and PR3-ANCA reliably distinguish GPA from IgA vasculitis and redirect management toward induction immunosuppression.

**Conclusion:**

Early dermatologic recognition, repeat biopsy with immunofluorescence at any clinical inflection point, and timely induction therapy can reverse otherwise life-threatening alveolar hemorrhage in GPA.

## Introduction

Granulomatosis with polyangiitis (GPA), formerly Wegener's granulomatosis, is a rare ANCA-associated vasculitis of small- to medium-sized vessels that classically involves the upper airway, lungs, and kidneys but may affect virtually any organ, including the skin^[^1,2^]^. Cutaneous manifestations are common and protean, ranging from palpable purpura to necrotizing ulcers, and frequently overlap with other small-vessel vasculitides – most notably IgA vasculitis (Henoch-Schönlein purpura) – when biopsy demonstrates leukocytoclastic vasculitis without confirmatory direct immunofluorescence (DIF)^[^3^]^.

HIGHLIGHTS

- Cutaneous ulcers may be the first clue to systemic granulomatosis with polyangiitis.
- Misdiagnosis as IgA vasculitis delayed recognition of underlying ANCA vasculitis.
- Strong c-ANCA (PR3) positivity and a skin biopsy confirmed granulomatosis with polyangiitis.
- Early immunosuppressive therapy reversed pulmonary hemorrhage and healed vasculitic ulcers.
- Dermatological evaluation is essential for the timely diagnosis of systemic vasculitis.

We describe a 47-year-old woman who was initially treated for IgA vasculitis for 1 year. Recurrent necrotizing ulcers, ENT involvement, and pulmonary hemorrhage – supported by strong PR3-ANCA positivity and a pauci-immune repeat biopsy – established the diagnosis of GPA. The case is reported in line with the CARE checklist for case reports^[^4^]^.

This diagnostic overlap is the principal pitfall illustrated in the present case. When an initial biopsy is reported as “leukocytoclastic vasculitis” without DIF, an erroneous label of IgA vasculitis can persist until catastrophic systemic features – such as alveolar hemorrhage – force reassessment. As untreated GPA carries an extremely poor prognosis^[^1,2^]^, recognizing the cutaneous-systemic pattern, repeating biopsy with immunofluorescence, and obtaining ANCA serology in a timely manner are the steps on which prognosis depends. Detailed epidemiological data and the comparative spectrum of GPA versus IgA vasculitis are addressed in the Discussion section.

## Case presentation

A 47-year-old woman presented to Lady Reading Hospital, Peshawar, in June 2025 with a 4-month history of painful, nonhealing ulcers over both legs, intermittent fever, and a 2-week history of hemoptysis. One year earlier, she had developed palpable purpura and erythematous plaques on the lower limbs. A skin biopsy at that time demonstrated leukocytoclastic vasculitis with predominant neutrophilic infiltration; however, DIF was not performed. On the basis of the clinical pattern of palpable purpura, together with the leukocytoclastic histology, a provisional diagnosis of IgA vasculitis was made. A short course of systemic corticosteroids produced only partial improvement, and the lesions subsequently recurred and evolved into deeper ulcers.

During the current illness, she reported recurrent nasal obstruction, intermittent epistaxis, and right-sided otalgia with purulent discharge. She denied weight loss, arthralgia, visual disturbances, or urinary symptoms. Her medical history included treated abdominal tuberculosis (2020) and chronic hepatitis C with confirmed sustained virologic response (2019). She was a non-smoker with no occupational toxin or illicit drug exposure.

### Timeline

A summary of the patient’s clinical course, from the initial cutaneous presentation through diagnosis and induction therapy, is shown in Table 1, in line with the CARE checklist.

**Table 1:** Chronological timeline of the patient’s illness, diagnostic workup, and treatment.

Date/Interval	Clinical event
2019	Hepatitis C eradicated; sustained virologic response confirmed.
2020	Treated for abdominal tuberculosis (full course completed).
~ 12 months before admission	Onset of palpable purpura and erythematous plaques on lower limbs.
~ 12 months before admission	First skin biopsy: leukocytoclastic vasculitis with neutrophilic infiltrate (direct immunofluorescence not performed). Provisional diagnosis: IgA vasculitis. Short course of systemic corticosteroids → partial improvement.
~ 6 months before admission	Recurrence of cutaneous lesions; gradual evolution into deeper, nonhealing ulcers.
~ 4 months before admission	Persistent painful ulcers over both legs; intermittent fever.
~ 2–3 months before admission	New-onset nasal obstruction, intermittent epistaxis, right-sided otalgia with otorrhea.
~ 2 weeks before admission	Onset of hemoptysis.
Day 1 (admission, June 2025)	Lady Reading Hospital, Peshawar. Hb 9.8 g/dL; urinalysis: microscopic hematuria; c-ANCA/PR3 > 100 U/mL; p-ANCA/MPO and ANA negative.
Day 1–2	HRCT chest: bilateral ground-glass opacities with crazy-paving pattern – diffuse alveolar hemorrhage. Paranasal sinus CT: mucosal thickening only.
Day 2	Repeat skin biopsy from active ulcer edge: pauci-immune necrotizing leukocytoclastic vasculitis; DIF negative for IgA, IgG, IgM, C3.
Day 2–4	IV methylprednisolone 1 g/day × 3 days.
Day 4 onward	Oral prednisolone 1 mg/kg/day; IV cyclophosphamide 15 mg/kg every 3 weeks (induction); TMP-SMX prophylaxis; transfusion support.
Day 14	Hemoptysis and fever resolved; inflammatory markers declining; ulcers granulating; discharged on tapering corticosteroids with planned maintenance immunosuppression and follow-up.

### Physical examination

On admission, the patient was pale but hemodynamically stable. Cutaneous examination revealed multiple punched-out ulcers on the anterior shins, 2–4 cm in diameter, with irregular, indurated borders, necrotic bases, and surrounding hyper- and hypopigmentation (Fig. 1).

**Figure 1.**
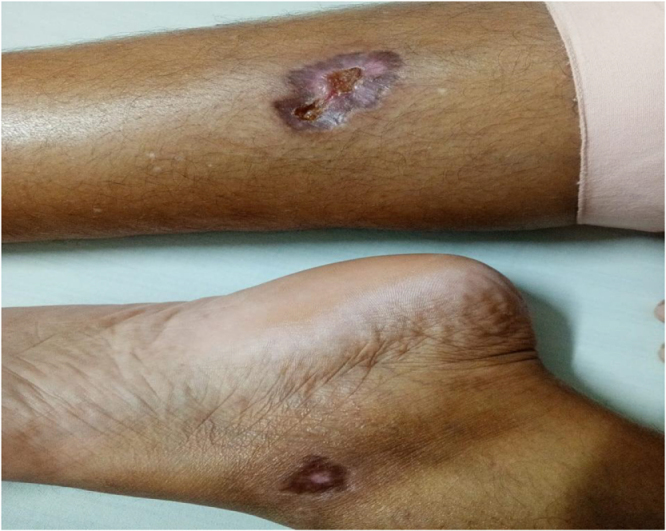
*Cutaneous findings of granulomatosis with polyangiitis:* multiple irregular, punched-out ulcers with necrotic bases and hyperpigmented margins over the lower limbs and dorsal feet.

Scattered purpuric macules and vasculitic nodules were present on the dorsal hands and feet (Figs 2 and 3), and widespread nonblanching purpura with ulcerated lesions involved the lower back and gluteal region (Fig. 4).

**Figure 2.**
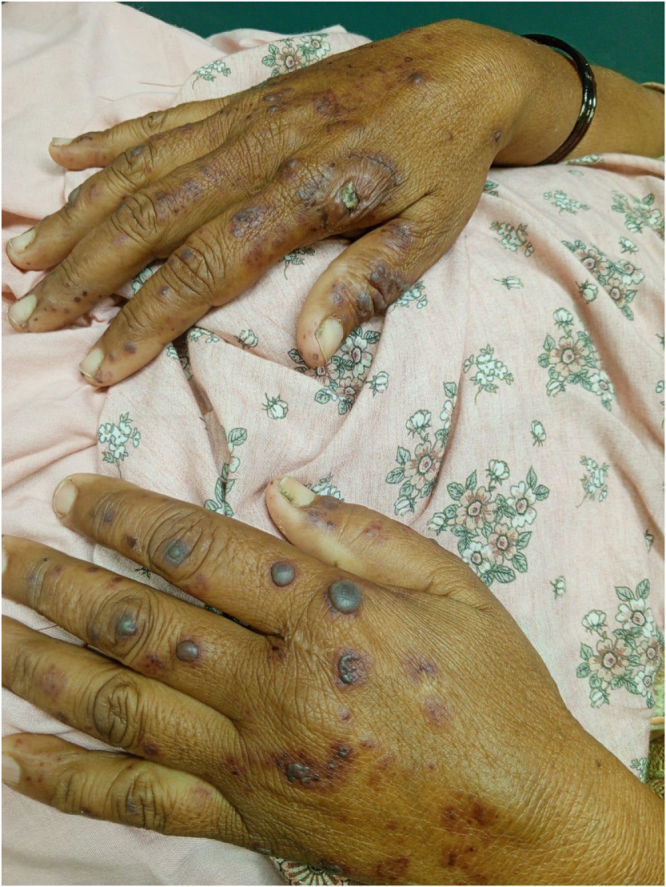
Cutaneous vasculitic nodules and ulcers over the dorsal hands.

**Figure 3.**
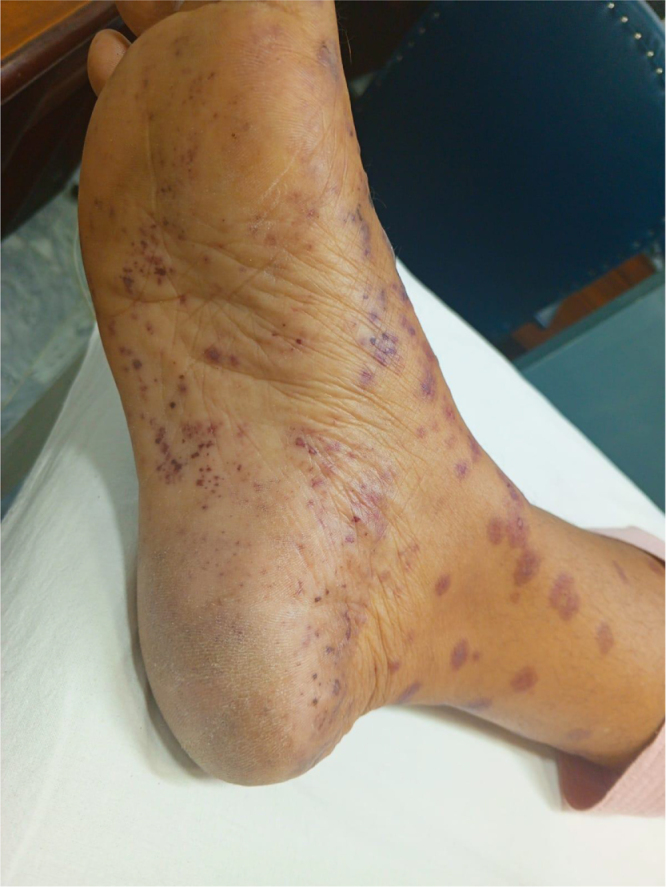
Palpable purpura on the plantar aspect of the foot.

**Figure 4.**
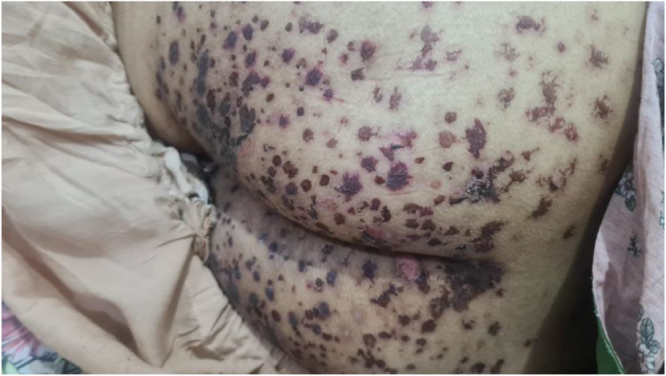
Extensive nonblanchable purpura and ulcerated lesions over the lower limb and buttock region.

There was no livedo or digital gangrene. Otoscopy showed a healed right tympanic membrane perforation; nasal mucosa demonstrated mild crusting without septal perforation. Bilateral coarse crackles were heard on chest auscultation. Cardiovascular and neurological examinations were unremarkable.

### Laboratory investigations

Hemoglobin was found to be 9.8 g/dL, with a leukocyte count of 8.5 × 10^9^/L. Urinalysis showed microscopic hematuria (8–10 RBCs/HPF) and trace proteinuria. The c-ANCA (anti-PR3) was strongly positive at >100 U/mL, while p-ANCA (anti-MPO) and ANA were negative. Liver enzymes were normal, and HCV RNA was undetectable. Screening for HIV, syphilis, and cryoglobulins was negative.

High-resolution CT of the chest demonstrated bilateral ground–glass opacities with a “crazy-paving” pattern and upper-lobe fibrotic bands – findings consistent with diffuse alveolar hemorrhage (Fig. 5). No cavitary nodules were identified. Paranasal sinus CT showed mucosal thickening without bony erosion. Bronchoalveolar lavage was not performed; the diagnosis of diffuse alveolar hemorrhage was made on the basis of new-onset hemoptysis, a fall in hemoglobin, and the characteristic HRCT pattern in the context of strongly positive PR3-ANCA, and induction immunosuppression was prioritized given the rapid clinical evolution.

**Figure 5.**
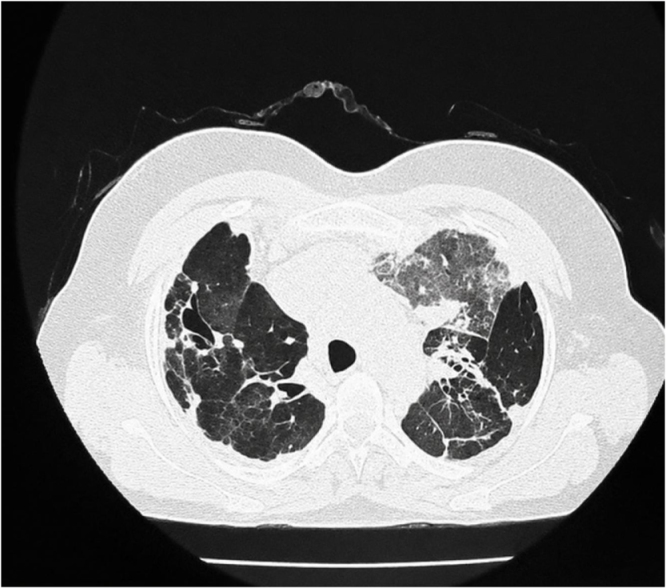
High-resolution CT chest shows ground–glass opacities with a “crazy paving” pattern and fibrotic upper-lobe changes.

### Histopathology

A repeat biopsy from the active ulcer edge demonstrated fibrinoid necrosis of small- to medium-sized dermal vessels with neutrophilic infiltration and leukocytoclasia, consistent with necrotizing leukocytoclastic vasculitis. No well-formed granulomas or infectious organisms were identified; PAS, Ziehl-Neelsen, and Grocott stains were negative. DIF was pauci-immune, with no IgA, IgG, IgM, or C3 deposition. The absence of IgA on DIF – in contrast to the unexamined index biopsy – was decisive in excluding IgA vasculitis.

### Diagnosis and management

The combination of recurrent necrotizing cutaneous vasculitis, ENT involvement, diffuse alveolar hemorrhage, strongly positive c-ANCA/PR3, and pauci-immune leukocytoclastic vasculitis on repeat biopsy established a diagnosis of GPA with cutaneous, upper airway, and pulmonary involvement. Renal involvement was limited to microscopic hematuria without overt glomerulonephritis.

### Treatment & follow-up

Induction therapy comprised pulse methylprednisolone 1 g IV daily for 3 days, followed by oral prednisolone 1 mg/kg/day, together with IV cyclophosphamide 15 mg/kg every 3 weeks. Trimethoprim-sulfamethoxazole prophylaxis for *Pneumocystis jirovecii*, calcium and vitamin D supplementation, and local wound care were provided. Anemia related to alveolar hemorrhage was managed with transfusion support. Within 2 weeks, hemoptysis and fever had resolved, inflammatory markers were declining, and ulcer bases had begun to granulate. The patient was discharged on a tapering corticosteroid regimen and scheduled for maintenance immunosuppression, serial ANCA monitoring, renal surveillance, and chest follow-up imaging.

## Discussion

Cutaneous involvement in GPA is reported in approximately 30–60% of patients during the course of the disease^[^3,5^]^. The dermatologic spectrum is wide: palpable purpura – typically on the lower extremities – is the most frequent finding, but ulcers, subcutaneous nodules, papulopustular eruptions, vesiculobullous lesions, digital gangrene, livedo reticularis, and pyoderma gangrenosum-like ulcerations all occur^[^5,6^]^. Deep necrotizing ulcers, as in our patient, are recognized but uncommon and are particularly prone to misdiagnosis. Multiple lesion types may coexist, and skin involvement may parallel internal disease severity, with vasculitic purpura associated with a higher likelihood of concurrent renal involvement^[^5^]^.

Cutaneous lesions can precede or coincide with systemic features and may be the sentinel clue to GPA^[^6^]^. In our patient, recurrent vasculitic ulcers were the earliest manifestation but were initially attributed to IgA vasculitis. The time from symptom onset to definitive diagnosis is frequently prolonged in GPA – often more than a year^[^5^]^ – and, during that interval, ongoing granulomatous inflammation can produce irreversible organ injury.

The diagnostic dilemma in this case centered on distinguishing GPA from IgA vasculitis. Both can present with palpable purpura and a leukocytoclastic small-vessel vasculitis on light microscopy. The decisive distinction is immunopathological: IgA vasculitis is defined by IgA-dominant immune-complex deposition on DIF, whereas GPA characteristically shows a pauci-immune pattern^[^7^]^. In our patient, DIF was not performed on the index biopsy, and the IgA vasculitis label persisted on clinical grounds alone for 1 year. The error was corrected only when the repeat biopsy demonstrated pauci-immune histology and PR3-ANCA returned strongly positive. PR3-ANCA is highly specific for GPA and positive in up to 90% of patients with active generalized disease^[^1,2^]^; its discovery, together with the negative immunofluorescence, was the turning point. Similar misdiagnoses have been reported, including a child with GPA initially treated as IgA vasculitis^[^7^]^ and an adult presenting with bullous purpura in whom ENT involvement and PR3-ANCA redirected the diagnosis^[^8^]^. GPA should therefore remain in the differential of any leukocytoclastic vasculitis with systemic features, particularly in adults, and DIF should be obtained on every initial biopsy.

Misdiagnosis of GPA is not limited to IgA vasculitis. Necrotic ulcers may be mistaken for chronic infectious ulcers – including cutaneous tuberculosis, non-tuberculous mycobacteria, deep fungal infection, or Nocardia – and are only excluded after extensive cultures and tissue stains^[^6^]^. Pyoderma gangrenosum is a further mimic; it produces painful undermined ulcers with neutrophilic infiltrate but lacks true vessel-wall vasculitis, and patients initially labeled as idiopathic pyoderma gangrenosum have later been found to have GPA when pulmonary or renal disease emerged^[^6^]^. In our patient, the demonstration of true vessel-wall inflammation on repeat biopsy, together with multiorgan involvement and PR3-ANCA, strongly favored GPA over both infectious mimics and pyoderma gangrenosum.

Tissue biopsy remains a cornerstone of GPA diagnosis. The classical histopathologic triad of necrotizing vasculitis, granulomatous inflammation, and tissue necrosis^[^9^]^ is rarely captured in full on a single skin biopsy, which most often shows leukocytoclastic vasculitis with fibrinoid necrosis. In a clinicopathologic study of 75 skin biopsies from GPA patients, 31% showed leukocytoclastic vasculitis, and only 19% showed granulomatous inflammation, with overt granulomatous vasculitis being rare in skin^[^10^]^. Therefore, a skin biopsy may confirm small-vessel vasculitis without demonstrating granulomas; nonetheless, in the appropriate clinical and serological context – as in our case – pauci-immune leukocytoclastic vasculitis with positive PR3-ANCA is essentially diagnostic. Where feasible, biopsy of other involved organs (lung, kidney, and ENT) increases diagnostic yield^[^9^]^.

Outcomes in GPA have transformed since the introduction of cyclophosphamide and corticosteroids: untreated, generalized GPA carried a 2-year mortality approaching 90%, whereas modern induction regimens achieve remission in approximately 90% of patients, with 5-year survival now exceeding 80%^[^11,12^]^. Relapses, however, occur in 30–50% of patients^[^13^]^. Rituximab is now considered at least equivalent to cyclophosphamide for induction in severe GPA and is often preferred for its more favorable toxicity profile; maintenance therapy typically employs azathioprine, methotrexate, or scheduled rituximab. Diffuse alveolar hemorrhage, as in our patient, is a life-threatening manifestation requiring prompt high-dose corticosteroids and cyclophosphamide (or rituximab); in our case, hemoptysis and the radiologic alveolar hemorrhage pattern reversed within 2 weeks of induction, mirroring outcomes reported when GPA is recognized and treated early^[^6^]^.

## Conclusion

This case carries three principal lessons. First, GPA can convincingly mimic IgA vasculitis when an initial skin biopsy demonstrates leukocytoclastic vasculitis without DIF; in any adult with this pattern, IgA vasculitis must remain a diagnosis of exclusion until DIF and ANCA testing have been obtained. Second, persistent or recurrent cutaneous lesions – particularly necrotizing ulcers and palpable purpura – are not merely a dermatologic concern but a window into systemic vasculitis, and re-biopsy with immunofluorescence at any clinical inflection point can be diagnostically decisive. Third, once GPA was recognized and induction therapy with pulse methylprednisolone and cyclophosphamide was initiated, our patient’s diffuse alveolar hemorrhage – an otherwise life-threatening complication – reversed within 2 weeks. Early dermatologic recognition, therefore, translates directly into the reversibility of severe organ involvement.

## Data Availability

Not applicable.

## Reporting checklist

This case report follows the CARE Checklist for Case Reports.
